# Study on Formulation, *in vivo* Exposure, and Passive Targeting of Intravenous Itraconazole Nanosuspensions

**DOI:** 10.3389/fphar.2019.00225

**Published:** 2019-03-28

**Authors:** Qi Yuan, Yanling Wang, Rufeng Song, Xianqiao Hou, Keke Yu, Jiaojiao Zheng, Juanmei Zhang, Xiaohui Pu, Jihong Han, Lanlan Zong

**Affiliations:** ^1^ School of Pharmacy, Institute of Materia Medica, Henan University, Kaifeng, China; ^2^ School of Pharmacy, The Institute for Science and Technology in Medicine, Keele University, Staffordshire, United Kingdom

**Keywords:** itraconazole nanosuspension, process optimization, *in vivo* pharmacokinetics, tissue distribution, passive targeting

## Abstract

The pharmacokinetic profile of a drug can be different when delivered as a nanosuspension compared with a true solution, which may in turn affect the therapeutic effect of the drug. The goal of this study was to prepare itraconazole nanosuspensions (ITZ-Nanos) stabilized by an amphipathic polymer, polyethylene glycol-poly (benzyl aspartic acid ester) (PEG-PBLA), by the precipitation-homogenization, and study the pharmacokinetic profile of the ITZ-Nanos. The particle size and morphology of nanosuspensions were determined by Zetasizer and field emission scanning electron microscope (SEM), respectively. The dissolution profile was evaluated using a paddle method according to Chinese Pharmacopoeia 2015. The level of ITZ in plasma and tissues was measured by a HPLC method. The optimized ITZ-Nanos had an average particle size of 268.1 ± 6.5 nm and the particles were in a rectangular form. The dissolution profile of ITZ-Nanos was similar to that of commercial ITZ injections, with nearly 90% ITZ released in the first 5 min. The ITZ-Nanos displayed different pharmacokinetic properties compared with the commercial ITZ injections, including a decreased initial drug concentration, increased plasma half-life and mean residence time (MRT), and increased concentration in the liver, lung, and spleen. The ITZ-Nanos can change the *in vivo* distribution of ITZ and result in passive targeting to the organs with mononuclear phagocyte systems (MPS).

## Introduction

Itraconazole (ITZ) is a wide-spectrum triazole antifungal agent that can be widely used in the treatment of both local and systemic fungal infections (Beule and Gestel, 2001; Boogaerts and Maertens, 2001). Its antifungal activities are dose-dependent, which means microorganisms that are resistant to the drug at a low dose may become susceptible when the dose is increased high enough (Rex et al., 1997). Originally, the drug was formulated and marketed as capsules and later developed as a liquid using Hydroxypropyl-β-cyclodextrin (HP-β-CD) as a solubilizer to overcome the poor bioavailability of the capsules (Glasmacher and Prentice, 2006). Due to toxicities associated with HP-β-CD (Rabinow et al., 2007), alternative formulations that can deliver a high dose safely without HP-β-CD have been explored. Nanosuspensions have been used to deliver the poorly soluble ITZ using various stabilizers, such as poloxamer 407 (Matteucci et al., 2006), Hydroxypropyl methyl cellulose (HPMC) (Matteucci et al., 2007; Matteucci et al., 2009), Polyvinyl alcohol and glycyrrhizinic acid (Fernández-Ronco et al., 2015), poloxamer 188 and sodium deoxycholate (Rabinow et al., 2007), tween 80 (Wlaz et al., 2015), the hybrid of HPMC and sodium dodecylbenzene sulfonate (SDS) (Azad et al., 2016), and tween 20 (Foglio Bonda et al., 2016). As many of these formulations have various limitations such as large particle size, poor stability, or toxicity for intravenous administration (Decuzzi et al., 2010; Pu et al., 2012), a new stabilizer, polyethylene glycol-poly(Benzyl aspartic acid ester) (PEG-PBLA), has been employed to stabilize ITZ nanoparticles (Zong et al., 2017). The PEG-PBLA-stabilized ITZ nanosuspensions (ITZ-Nanos) were fabricated by a combination of microprecipitation and high-pressure homogenization (mPHPH) method (Zong et al., 2017). The ITZ-Nanos demonstrated good stability and biocompatibility in comparison with poloxamer 188-stabilized nanosuspensions. It also showed promising antifungal effects.

There has been report that nanoparticles may alter the pharmacokinetic profile and tissue distribution of a drug and improve its efficacy (Rabinow et al., 2007; Chiang et al., 2014; Sigfridsson et al., 2017; Li et al., 2018). In this work, the various factors affecting the preparation of the ITZ-Nanos will be evaluated and optimized first, and then their physicochemical characteristics were investigated, such as particle size, morphology, and *in vitro* dissolution, followed by evaluation of the pharmacokinetic profile and tissue distribution of the ITZ-Nanos in comparison to the commercial injections in rats and mice. As a result, it was found that ITZ-Nanos can change the biodistribution of ITZ and accumulate in MPS organs more than ITZ injections. This performance could be an advantage to treat fungus infection in these organs and tissues and to reduce the side effect of ITZ to other parts of the body (Cao et al., 2018).

## Materials and Methods

### Materials

PEG-PBLA was synthesized in our own laboratory, and the details of the methods can be found elsewhere (Zong et al., 2017). Itraconazole was bought from Zhejiang Huafang Pharmaceutical Co., Ltd. (Zhejiang, China). Methanol (HPLC grade) was gained by Tianjin Concord Technology Co., Ltd. (Tianjin, China). ITZ injections were the product of Xian Janssen Pharmaceutical Ltd. (Xian, China). All other reagents were analytical grade.

### Process Optimization of ITZ-Nanos

ITZ-Nanos were prepared by microprecipitation-homogenization method. Firstly, equal amount of ITZ and PEG-PBLA were completely dissolved in moderate amount of dimethyl sulfoxide (DMSO) at 60°C to form a 50 mg/ml ITZ solution (the good solvent phase). In order to obtain a coarse suspension, this solution was added to the 50 ml of water (antisolvent phase), which was agitated at 1,500 rpm using a magnetic stirrer at room temperature. The suspension was continuously stirred for 10 min and subsequently homogenized using a NS1001L2K high-pressure homogenizer (Niro Soavi S.p.A. Co, Parma, Italy). The homogenization process consisted of a preliminary homogenization stage of 2 cycles at 200 bar and another 2 cycles at 500 bar, followed by a main homogenization stage at predetermined number of cycles at a pressure of 800, 1,000, or 1,200 bar, as indicated in the Results, to obtain the ITZ-Nanos. The nanosuspensions were frozen at −20°C for 12 h in a refrigerator after 5% (Mannitol, w/v) were added and subsequently lyophilized at −50°C for 36 h under vacuum using a freeze dryer to obtain a dry powder.

### Particle Size and Zeta Potential

ITZ-Nanos were first reconstructed in water for injection to gain a liquid dispersion. To observe the physicochemical properties of the dispersion, a Malvern Zetasizer Nano ZS90 in the light of Dynamic Light Scattering (DLS) were used. Repeats of measurement and the average were taken for each sample.

### Particle Morphology

A scanning electron microscope (SEM) was used to observe the dimension and morphology of particles. Freshly prepared samples were sprayed onto SEM specimen holders and coated with gold/palladium using a sputter coater after all water was removed under ambient conditions. Photomicrographs were taken using a JSM-7100F field emission SEM (JEOL Co., Japan) with an accelerating voltage of 10 kV.

### *In vitro* Dissolution

The dissolution curve of the ITZ-Nanos *in vitro* was studied using a ZRS-8G dissolution test analyzer by the paddle method according to Chinese Pharmacopeia 2015. In a brief, samples containing equivalent of 10 mg ITZ were added to 500 ml PBS (pH 7.4) containing 0.5% (w/v) SDS at 37°C, and the rotation speed of the paddle was set at 75 rpm. At a scheduled time of 2, 5, 15, 30, 45, 60, and 120 min, 5 ml of the dissolution medium was withdrawn and 5 ml of fresh medium was compensated immediately to maintain the sink condition following each sampling. Samples were filtrated through 0.45 μm filters and analyzed with HPLC. All experiments were performed in triplicate.

### *In vivo* Pharmacokinetics of ITZ-Nanos

All animal experiments were approved by the Ethics Committee on Animal Experiment of Henan University, and toxic effect was not emerged from all the rats. Adult male Sprague Dawley rats, weighing about 190–210 g, were fed in rooms at controlled temperature and relative humidity for at least 1 week prior to pharmacokinetic test. The rats were fasted for 12 h before experiments and were randomly and equally divided into two groups. ITZ injections or ITZ-Nanos were injected *via* tail vein at a dose of 15 mg/kg. Blood samples (0.5 ml) were collected in heparinized tubes at 0, 0.033, 0.083, 0.25, 0.5, 1, 2, 4, 6, 8, and 10 h after administration. After immediate centrifugation, plasma samples were stored at −20°C until further analysis.

### Tissue Distribution of ITZ-Nanos

Male Kunming species mice weighing 19–21 g were acclimatized for at least 1 week prior to experiment, fed with standard diet, and permitted water ad libitum. All animal experimentations were evaluated and approved by the Ethics Committee on Animal Experiment of Henan University, and toxicity did not appear in all mice. The mice were randomly divided into two groups. ITZ injections and ITZ-Nanos were respectively administered intravenously at a dose of 15 mg/kg *via* the tail vein with a 1 ml syringe coupled with a 28G1/2 needle. At scheduled time (0.25, 2, and 4 h), five mice from each group were anesthetized by ethyl ether and sacrificed by cervical dislocation. The liver, kidney, heart, lung, and spleen were resected and washed with cold saline solution (0.9% NaCl) to remove surface blood and then quick dried with tissue paper. An additional incision was made in the heart to allow the blood remained in the cardiac atria and ventricles squeezed out. After collection, tissue samples were stored at −20°C until further analysis.

### High-Performance Liquid Chromatography

ITZ concentrations in dissolution medium were determined using a Waters 2695 HPLC system (Waters Corporation, Milford, USA) equipped with an automatic sampler and a variable wavelength UV detector set at 263 nm. A RP-C_18_ chromatographic column (5 mm, 250 × 4.6 mm, Thermo Syncronis, Waltham, USA) was utilized with the column temperature at 25°C. The mobile phase composed of methanol and water (85:15, v/v), and the flow rate was 1.0 ml/min. A 20 μl sample was injected into the column for analysis.

For the analysis of plasma samples, nimodipine (2.5 μg/ml) was used as an internal standard. About 100 μl of methanol and 200 μl of the internal standard solution were added to 100 μl of plasma, and the mixture was vortexed for 3 min. The samples were subsequently subjected to ultrasonication for 30 s and centrifuged at 13,000 g for 10 min. The supernatants were taken and dried under nitrogen atmosphere. The residuum was reproduced with 100 μl of methanol and injected into a HPLC column for analysis as described above. Tissue samples were weighed accurately and homogenized by a DY89-1 tissue homogenizer (Ningbo Scientz Biotechnology Co., Ltd. China) after the addition of 300 μl physiologic saline, which contains 10% (v/v) methanol. These samples were subsequently processed the same way as serum samples and analyzed by HPLC as mentioned above.

### Data Analysis

Statistical analysis was performed using two-tailed Student t-test. *In vivo* pharmacokinetic parameters were calculated with a DAS 2.0 pharmacokinetic program (Mathematical Pharmacology Professional Committee of China, Shanghai, China). Area under curve (AUC) in the tissue distribution test was calculated using the linear trapezoidal method, and the *C*_max_ values were observed from the curves in the tissue distribution experiment. To estimate the targeting efficiency of ITZ-Nanos, relative uptake rate (*R*_e_) and ratio of peak concentration (*C*_e_) were calculated by the following equations.

$$R_{e}={\left({\mathrm{AUC}}_{X}\right)}_{N}/{\left({\mathrm{AUC}}_{X}\right)}_{I}$$ (1)

$$C_{e}={\left(C_{\max}\right)}_{N}/{\left(C_{\max}\right)}_{I}$$ (2)

where AUC*_X_* is behalf of the area under time-concentration profile of No. *X* organ or tissue. The *N* and *I* represent ITZ-Nanos and ITZ injection, respectively.

## Results and Discussion

### Process Optimization for the Preparation of ITZ-Nanos

**Figure 1:**
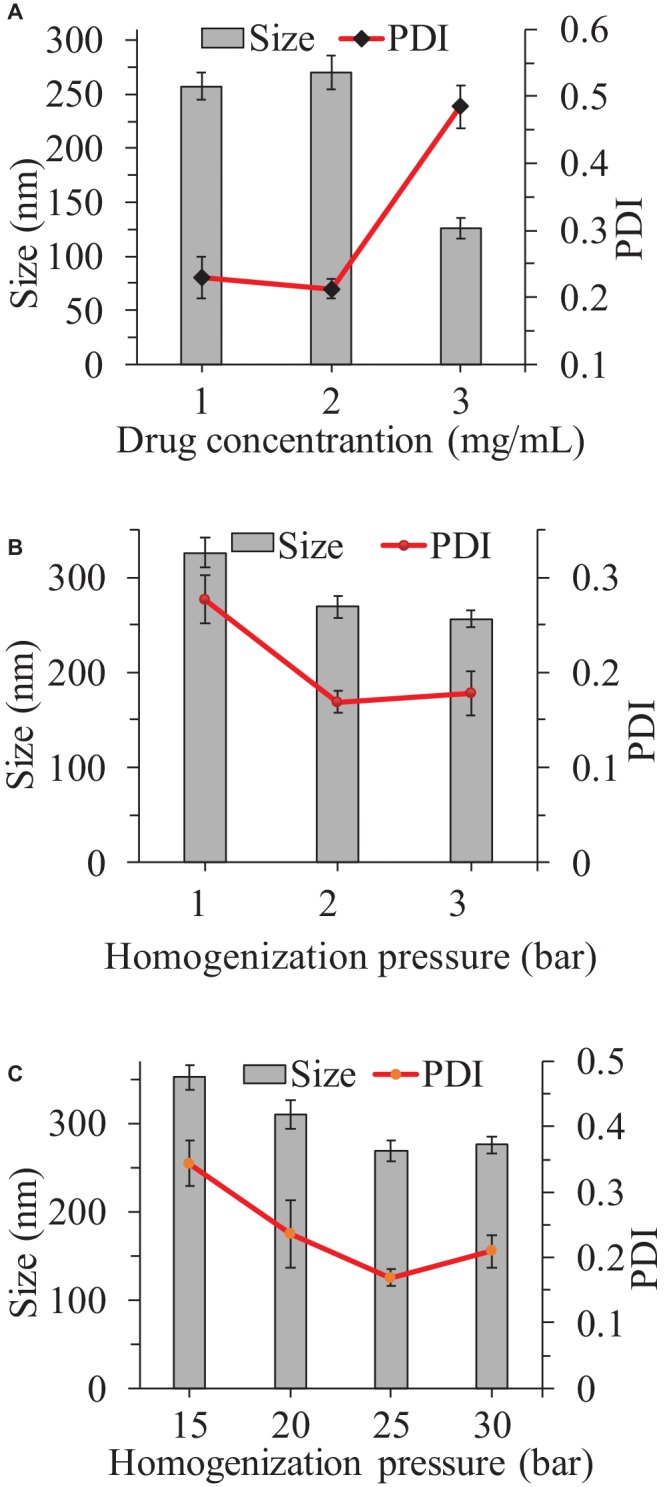
Influence of the drug concentration **(A)**, homogenization pressure **(B)**, and homogenization cycles **(C)** on the particle size reduction of ITZ-Nanos.

In this work, a microprecipitation-high-pressure homogenization process was used to obtain ITZ-Nanos. In order to find the optimum conditions for this process, the influences of drug concentration, homogenization pressure, and number of cycles on the particle dimension of the nanosuspensions were evaluated. While the other process parameters were fixed, the effect of drug concentration was evaluated in the range from 1 to 3 mg/ml as shown in Figure 1A. It can be seen that the particle size increased slightly when the concentration increased from 1 to 2 mg/ml and then decreased with a further increase of drug concentration to 3 mg/ml. The PDI remained virtually unchanged when the concentration increased from 1 to 2 mg/ml, but increased dramatically when the concentration increased from 2 to 3 mg/ml. Although the particle size was smallest at the 3 mg/ml, the PDI was largest, which was an indication that the physical stability was likely to be poor (Verma et al., 2011; Papdiwal and Pande, 2014; Ren et al., 2016; Guo et al., 2017). Therefore, 2 mg/ml was considered to be the ideal concentration as the particle sizes and PDIs were not considerably higher that of 1 mg/ml. It can be seen from Figure 1B that an increase of the homogenization pressure from 800 bar to 1,000 bar reduced the particle sizes and PDI substantially, but a further increase to 1,200 bar failed to reduce these any further. As the number of homogenization cycles increased, the particle size and PDI decreased first and then increased lightly, with the smallest particle size observed at 25 homogenization cycles (Figure 1C). Therefore, the conditions adopted were 2 mg/ml drug concentration, 1,000 bar pressure, and 25 cycles through the homogenizer.

### Particle Size, Zeta Potential, and Morphology

**Figure 2:**
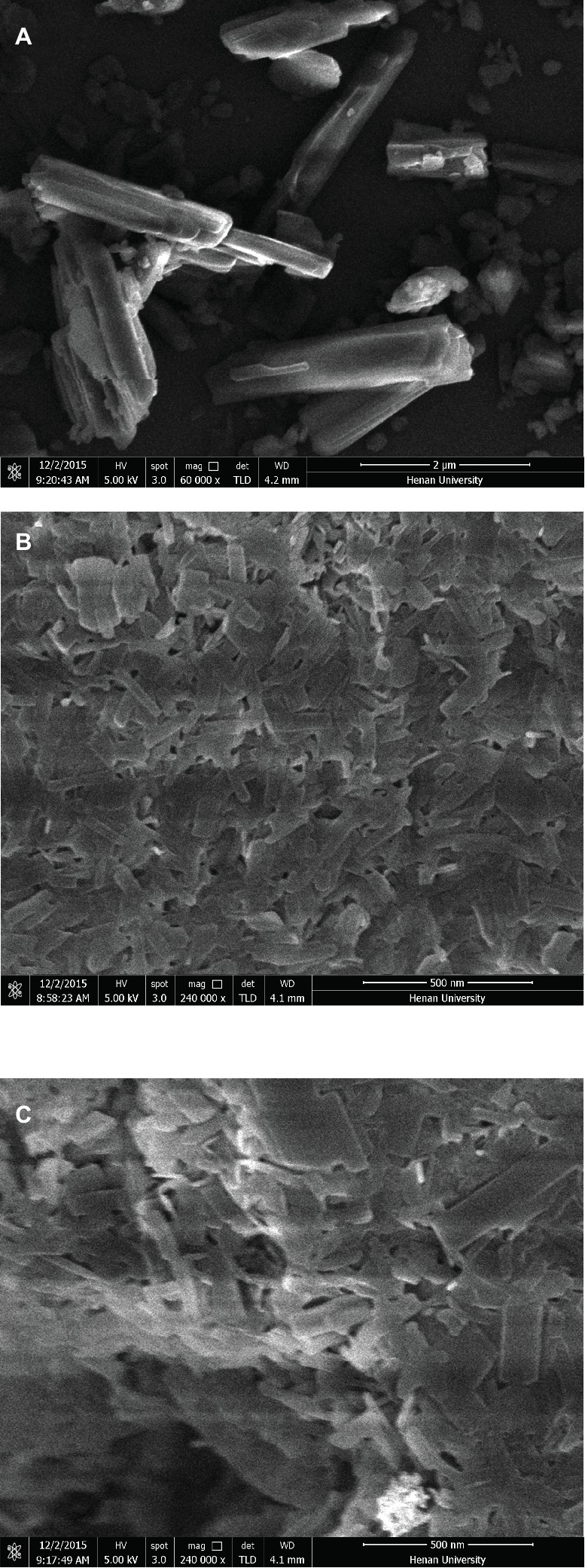
SEM images of **(A)** crude ITZ, **(B)** ITZ-Nanos before lyophilizing, and **(C)** ITZ-Nanos after lyophilizing.

As seen from Table 1, the average particle size, polydispersity index, and zeta potential did not change significantly after lyophilization. The lyophilized ITZ-Nanos presented a rectangular shape (Figure 2), different from the claviform exhibited by the bulk drugs, which is the same as those reported by Wong (Wong et al., 2006). However, the particle size of ITZ-Nanos was several times smaller compared with the bulk drugs (1–5 μm). The dimension of ITZ-Nanos measured by SEM (Figure 2) was conformed to that by DLS. It means that dimension of ITZ-Nanos in this research was close to ITZ nanosuspensions by Wong et al. (2006) but was far smaller than those by Rabinow et al. (2007). This implied that ITZ in the ITZ-Nanos could dissolve more quickly in blood and reduce its distribution in organs of the MPS, and consequently, the depot effect in MPS might not be as evident as those by Rabinow et al. (2007). Such potential effect can be ascribed to the effect of dimension and appearance on the *in vivo* performance of nanoparticles (Decuzzi et al., 2010).

**Table 1:** The mean particle sizes and zeta potentials of ITZ-Nanos.

Parameters	Before lyophilization	After lyophilization
Size (nm)	268.1 ± 6.5	289.5 ± 6.4
PDI	0.157 ± 0.019	0.187 ± 0.017
Zeta potential (mV)	−17.5 ± 0.8	−19.3 ± 1.8

### *In vitro* Dissolution

**Figure 3:**
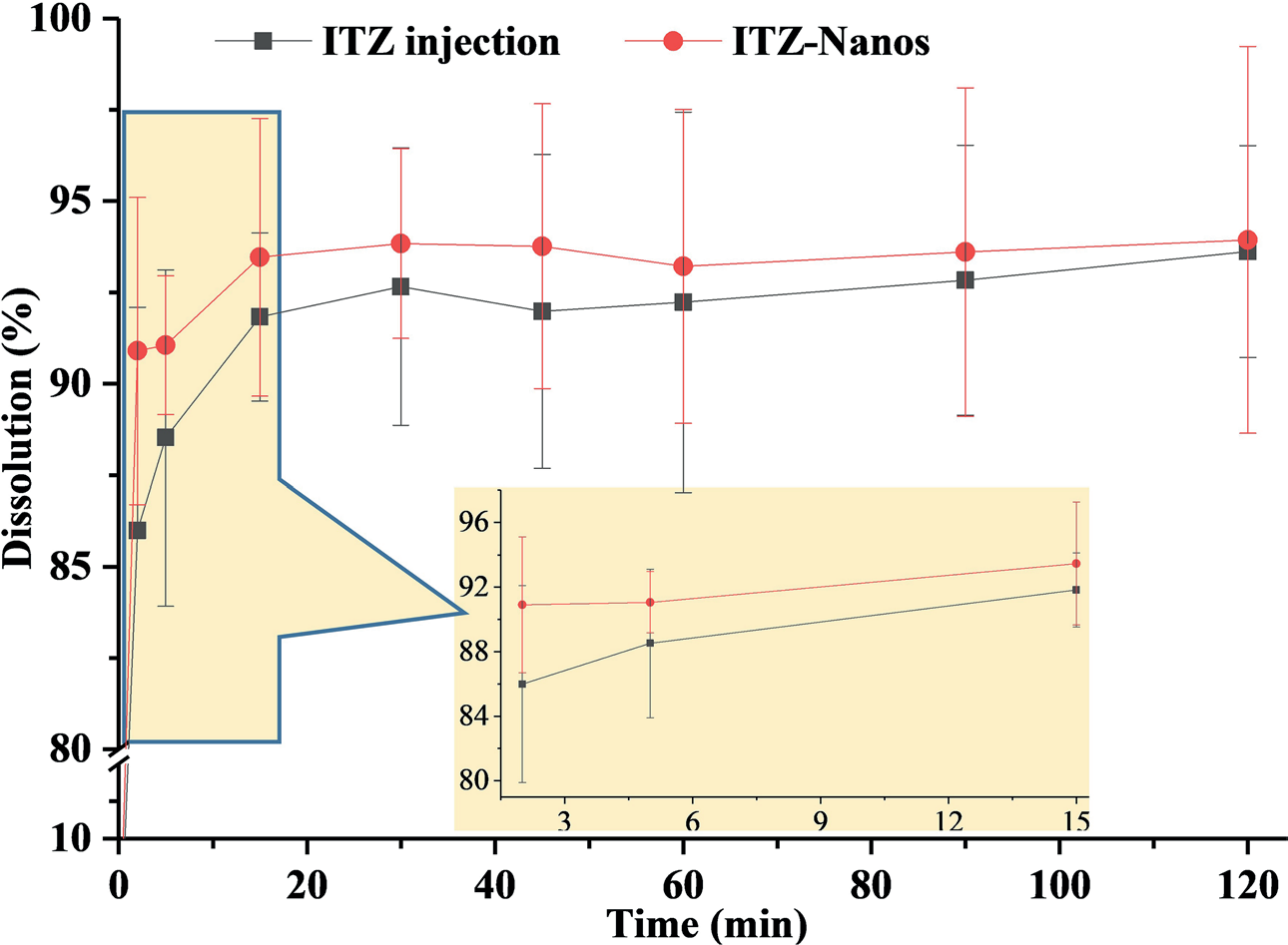
*In vitro* dissolution of ITZ injection and ITZ-Nanos.

The dissolution rate of ITZ-Nanos, in comparison with ITZ injections, was investigated, and the results were shown in Figure 3. The dissolution profile of ITZ from nanoparticles was essentially similar to that of the injections, with the accumulative dissolution rate of ITZ-Nanos being reached 86.0% within 2 min and ITZ injections being 91.0% within 2 min. The reasons for the fast dissolution of the ITZ-Nanos were considered to be mainly owing to the small dimension of the nanoparticles. Slow dissolution rate is one of the key issues affecting the clinical application of newly developed medicines that are poorly soluble in water, and reducing particle size has been one of the often adopted strategies to conquer the problem. The micronization of poorly soluble drugs, where particles are reduced to the micrometer range, improves dissolution rate because of the associated increase of surface area without actually increasing the solubility of the drug (Teeranachaideekul et al., 2008; Chavhan, 2013). However, when the particle size is decreased to the nanometer range, through formulation technologies, such as nanoemulsion (Bu et al., 2014; Yadav et al., 2016; Kaur et al., 2017), liposome (Koshkina et al., 2004), solid lipid nanoparticle (Baek and Cho, 2015), micelle (He et al., 2016; Chen et al., 2017), and nanosuspension (Ghosh et al., 2008; Maged et al., 2016; Yin et al., 2016), both the surface area and the solubility of the drugs are increased significantly, resulting in substantially increased dissolution rate. The particle sizes of the ITZ-Nanos were below 300 nm; therefore, both the total surface area and the solubility of the ITZ-Nanos would have been increased. Although the dissolution profiles of the ITZ-Nanos were similar to ITZ injections, its toxicity could be extremely reduced on account of the better biocompatibility of the stabilizer used in the nanosuspensions than that of cyclodextrin in the ITZ injections (Zong et al., 2017).

### *In vivo* Pharmacokinetics of ITZ-Nanos

**Figure 4:**
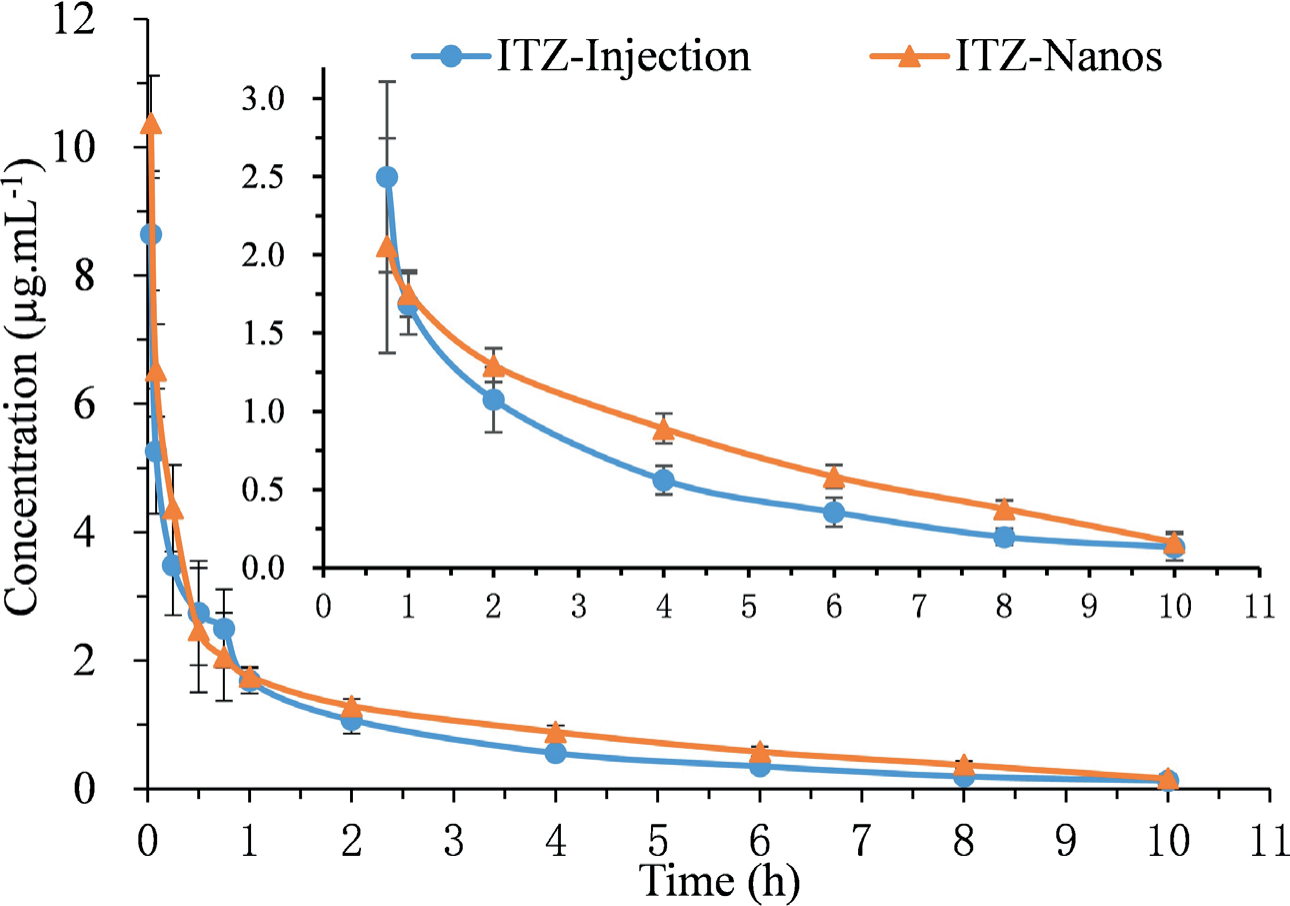
Mean plasma concentration-time curves of five rats after I.V. administration of ITZ-Nanos and ITZ injection at 15 mg/kg.

The blood concentration-time profiles of ITZ after I.V. administration are shown in Figure 4, and the relevant pharmacokinetic parameters obtained are listed in Table 2. The results indicated that ITZ-Nanos exhibited a slightly delayed clearance in the blood compared to ITZ injections. In addition, the MRT (2.92 ± 0.39 h) and the half-life (*t*_1/2_, 2.31 ± 0.25 h) of the ITZ-Nanos were longer than those observed of the ITZ injections (2.67 ± 0.22 h, 1.55 ± 0.08 h). This could be due to that ITZ-Nanos were partly recognized and captured by reticuloendothelial system (RES) and subsequently released back into the systemic circulation gradually (Peters et al., 2000; Pu et al., 2012). As reported in literatures, phagocytosis of nanoparticles by RES organs following I.V. injection may take from several minutes to hours, depending on the dimension and ingredient of the particle (Manjunath and Venkateswarlu, 2006). The ITZ-Nanos phagocytosed by RES might dissolve slowly in phagocytes and release slowly into blood circulation and maintain a blood level longer than ITZ injections. This way, the captured ITZ-Nanos by RES can serve as a drug depot slowly releasing drug into the systemic circulation to prolong drug action. This could be beneficial to certain drug classes, whose efficacy are enhanced by a prolonged therapeutic time and peak plasma concentration, such as the case of itraconazole. But the depot effect in this study was not as substantial as that reported in another study (Rabinow et al., 2007), probably due to the small particle size and fast dissolution of ITZ-Nanos.

**Table 2:** Comparison of the main PK parameters for two formations.

Parameter	ITZ injection	ITZ-Nanos
AUC_0–∞_ (mg/l × h)	10.01 ± 1.76	8.76 ± 1.42
MRT_0–∞_ (h)	2.67 ± 0.22	2.92 ± 0.39
V/(l/kg)	4.43 ± 0.48	5.13 ± 0.70
CL (l/h/kg)	1.50 ± 0.14	1.54 ± 0.09
*t*_1/2zeta_ (h)^*^	1.55 ± 0.08	2.31 ± 0.25

* Statistical significance (p < 0.01).

### Tissue Distribution of ITZ-Nanos

**Figure 5:**
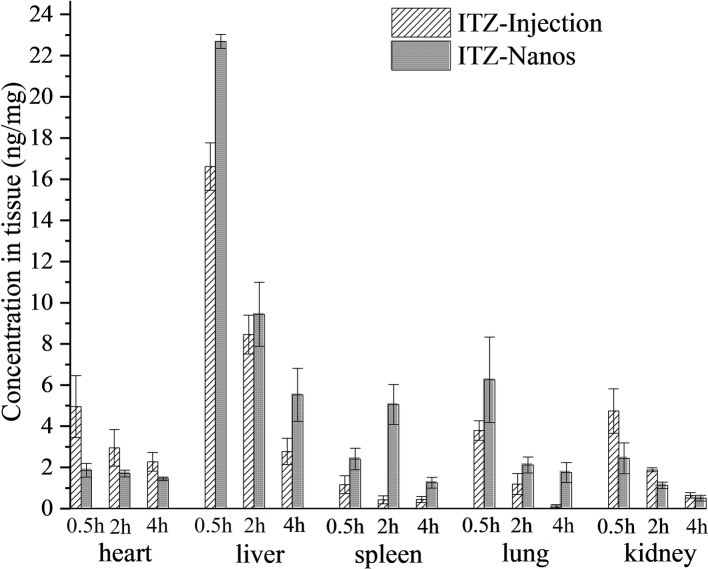
Tissues distribution of ITZ after I.V. administration of two formulations in mice (*n* = 5) at 0.25 h, 2 h, and 4 h.

Figure 5 shows the mean drug concentration with time in tissues after I.V. administration of the ITZ formulation to mice. The AUC_(0–4)_, *C*_max_, the *R*_e_, and *C*_e_ of the two formulations in the various tissues are shown in Table 3. It can be seen from Table 3 that the concentrations of ITZ in liver, lung, and spleen were remarkably higher for ITZ-Nanos compared to those of ITZ injections, and the *R*_e_ and *C*_e_ values in the three tissues were larger than those in other tissues, suggesting ITZ-Nanos passively targeted to these organs. This could be due to that ITZ-Nanos were recognized as foreign substances and quickly endocytosed by phagocytes of the mononuclear phagocyte system (MPS) in blood circulation (Pu et al., 2012). The results of tissue distribution of ITZ further confirmed that RES-related organs, containing liver, lung, and spleen, could potentially serve as a drug reservoir depot for prolonged effect. Similar consequences were also discovered by others for clofazimine, 10-HCPT, and curcumin nanosuspensions (Peters et al., 2000; Pu et al., 2012; Bi et al., 2017). Another noticeable result from Figure 5 and Table 3 is that the ITZ concentration in the kidney is lower for ITZ-Nanos compared with ITZ injections, which is a potential advantage of ITZ-Nanos in reducing the toxicity of the drug to kidney.

**Table 3:** The AUC_(0–*t*)_ and *C*_max_ in different tissues of mice (*n* = 5).

Tissues	AUC_(0–*t*)_ (ng·h/mg)		*R*_e_	*C*_max_ (ng/mg)		*C*_e_
	ITZ injection	ITZ-Nanos		ITZ injection	ITZ-Nanos	
Heart	11.92 ± 1.09	5.82 ± 0.49	0.49	5.45 ± 0.49	1.95 ± 0.21	0.36
Liver	30.58 ± 3.16	39.06 ± 4.12	1.28	21.04 ± 2.08	24.97 ± 2.38	1.19
Spleen	2.06 ± 0.18	12.61 ± 1.31	5.78	1.05 ± 0.12	5.34 ± 0.53	3.75
Lung	5.06 ± 0.49	9.44 ± 0.96	1.87	6.19 ± 0.57	7.15 ± 0.67	1.16
Kidney	7.49 ± 0.76	4.35 ± 0.45	0.58	6.15 ± 0.55	2.90 ± 0.32	0.47

## Conclusions

The effects of drug concentration, homogenization pressure, and number of cycles of homogenization were evaluated, and the optimal conditions were 2 mg/ml, 1,000 bar, and 25 times, respectively. The dimension of ITZ-Nanos did not alter considerably after lyophilization, and the ITZ in the nanosuspensions displayed a rectangular shape. The ITZ-Nanos had a similar dissolution profile to that of ITZ injection with about 90% of the drug released in the first 5 min. The results indicated that ITZ-Nanos could moderately prolong circulating time in plasma and increase exposure time in rats. It was found that ITZ-Nanos accumulated in MPS organs, such as liver, spleen, and lung, more than ITZ injections. This performance could be an advantage to treat fungus infection in these organs and tissues and to reduce the side effect of ITZ to other parts of the body. It remains future work to evaluate the implications of such changes on the antifungal activity *in vivo*.

## Data Availability

All datasets generated for this study are included in the manuscript.

## Author Contributions

QY and YW prepared a draft manuscript. RS, XH, and KY did most of experiments in this study. JZhe and JZha dealed with experiment data. JH and LZ revised the manuscript. XP provided funds and executed the project management.

### Conflict of Interest Statement

The authors declare that the research was conducted in the absence of any commercial or financial relationships that could be construed as a potential conflict of interest.
